# Corneal Characteristics After Small-Diameter DMEK Graft for Fuchs Corneal Dystrophy—Long-Term Observation

**DOI:** 10.3390/jcm14072185

**Published:** 2025-03-23

**Authors:** Anna Machalińska, Monika Kuśmierz-Wojtasik, Krzysztof Safranow, Magda Kossmann

**Affiliations:** 1 First Department of Ophthalmology, Pomeranian Medical University, 70-204 Szczecin, Poland; 2 Department of Biochemistry and Medical Chemistry, Pomeranian Medical University, 70-204 Szczecin, Poland

**Keywords:** small-diameter grafts, Fuchs corneal dystrophy, Descemet Membrane Endothelial Keratoplasty (DMEK), endothelial cell loss, best-corrected visual acuity, central corneal thickness, astigmatism reduction, higher-order aberrations, long-term follow-up

## Abstract

**Background/Objectives**: This study aimed to evaluate long-term postoperative outcomes following the use of small-diameter grafts in Descemet Membrane Endothelial Keratoplasty (DMEK) procedures. **Methods**: Thirty-four eyes were evaluated after DMEK surgery. Best-corrected visual acuity (BCVA), endothelial cell density (ECD), endothelial cell loss (ECL), central corneal thickness (CCT), mean keratometry (MK), mean astigmatism (MA), astigmatism asymmetry (AA), and higher-order aberrations (HOA) were assessed at baseline and 12, 24 and 36 months after surgery using anterior segment swept-source OCT (CASIA2, Tomey, Japan). **Results**: BCVA gradually improved during the 12-month follow-up, after which the stabilisation of this parameter was documented. Compared with the donor values, the cumulative median ECL reached approximately 63.95% over 36 months. No significant changes in total keratometry between baseline recordings and 36-month data were observed. Total astigmatism power significantly decreased between baseline and the 12th month and subsequently between the 12th and 24th month, with consecutive stabilisation of astigmatism power from the 24th month to the 36th month of follow-up. Significant reductions in HOA were observed until the 12th month, followed by the stabilisation of these parameters. **Conclusions**: The use of smaller grafts in DMEK demonstrates high effectiveness in maintaining high visual and refractive quality while offering potential advantages in tissues.

## 1. Introduction

Fuchs endothelial corneal dystrophy (FECD) is the most common corneal endothelial dystrophy and represents the leading indication for corneal transplantation worldwide. It is a complex and heterogeneous genetic disease characterised by the progressive decline of corneal endothelial cells (CECs) and the formation of extracellular matrix excrescences in Descemet’s membrane (DM), called guttae, which leads to corneal oedema and loss of vision [1]. Descemet membrane endothelial keratoplasty (DMEK) represents the technique of choice in the management of FECD. It allows the selective replacement of diseased or dysfunctional endothelium, resulting in anatomic and functional restoration of the cornea [2,3,4].

One of the major characteristics of FECD is the origin of changes in the central cornea. CECs are predominantly damaged in the centre, and as the disease progresses, the guttae radiate out towards the periphery [1]. Thus, small-diameter DMEK donor grafts could theoretically replace the centrally damaged cells, sparing the marginally dysfunctional and healthy peripheral endothelium. Indeed, a smaller DMEK graft has several advantages. First, graft detachment was greater in the group of patients who received large grafts. Notably, a larger graft size increases the rebubbling rate [5,6]. This may be due to the longer contact of the gas or air bubble with the whole graft area in smaller grafts. Second, small grafts represent the optimal solution in the case of peripheral tears of donor tissue, enabling successful preparation. If the donor button is torn, obtaining a standard diameter DMEK graft is not possible, and the tissue is usually discarded. These can be easily managed with a smaller graft size, avoiding tissue waste. Additionally, smaller grafts may offer better graft adherence due to a reduced surface area requiring attachment, potentially decreasing the risk of postoperative detachment. The lower level of peripheral graft manipulation may also contribute to reduced endothelial cell loss, preserving the viability of the transplanted cells. Furthermore, smaller graft diameters could be particularly beneficial for patients with anatomical constraints, such as a shallow anterior chamber or previous intraocular procedures that may complicate graft unfolding and positioning. Despite this, it may be presumed that a larger graft size may lead to a higher survival rate over time, considering the greater number of transplanted endothelial cells [6,7]. However, it seems reasonable to assume that a lower amount of transplanted tissue was associated with a lower risk of rejection.

Considering the above findings, we investigated the time course changes in corneal parameters during a three-year follow-up after DMEK surgery with the use of a small graft size. We also evaluated the incidence of the most common postoperative complications. To the best of our knowledge, this might be the first study to provide such a broad analysis of dynamic variations in corneal keratometry, astigmatism, astigmatism asymmetry, and HOA values based on anterior segment swept-source optical coherence tomography (SS-OCT) recordings in long-term observations.

## 2. Materials and Methods

Thirty-four eyes with FECD were retrospectively analysed post-DMEK. Inclusion criteria encompassed clinically confirmed FECD, the absence of other significant corneal pathologies, no advanced scarring in the anterior corneal layers, no severe corneal deformities, no active inflammation, and no severe dry eye syndrome. To prevent postoperative pupillary block, all patients underwent preoperative YAG laser iridotomy at the 6 o’clock position relative to the corneal limbus before surgery. All surgical procedures were performed by an experienced surgeon (AM) at the 1st Ophthalmology Department in Szczecin. To ensure a high graft survival rate, in accordance with previous studies, corneal flaps with an endothelial density of no less than 2800 cells/mm^2^ were used as the transplant material. The median donor ECD was 2945.50 cells/mm^2^. Before storage, the endothelium was evaluated using mirror microscopy (Konan CellCheck EB-10; Konan Medical USA Inc., Irvine, CA, USA). After tissues were obtained from donors via autopsy or multiorgan donation, they were placed in Eusol-C fluid (Alchimia, Ponte San Nicol, Italy) and stored at 2–6 °C at the West Pomeranian Eye Tissue Bank in Szczecin for no longer than 14 days.

The graft flap was prepared immediately before the transplantation procedure in the operating room. All grafts were prepared and placed on a natural medium immersed in a 0.06% solution of trypan blue dye (Vision Blue, Zuidland, Netherlands; D.O.R.C.). The surgeon then excised the grafts to the desired diameter (median = 6.5 mm, IQR = 0.25 mm), using a Hessburg–Barron trephine (Barron Precision Instruments, Grand Blanc, MI, USA). The graft diameter was selected to ensure optimal adaptation to the anatomical dimensions of both the donor and recipient cornea, facilitating proper graft positioning and adherence. The DMEK procedure involved the selective replacement of the recipient’s endothelium and Descemet’s membrane with anatomically corresponding donor tissues. All procedures were performed under local anaesthesia. The surgery commenced with a 2.4 mm corneal incision, followed by the filling of the anterior chamber with viscoelastic to maintain anterior segment stability. The recipient’s Descemet’s membrane and dysfunctional endothelium were meticulously stripped using a Price–Sinskey scraper. The descemetorhexis area was 0.5 mm–1 mm larger than the diameter of the donor graft, optimising graft adherence and positioning within the recipient’s corneal bed. After the procedure, the viscoelastic was rinsed from the anterior chamber. The Descemet’s membrane was carefully separated from the corneal stroma of the donor and stained. A flap of the appropriate diameter was excised using a trephine, immersed in sterile balanced salt solution, and placed in a dedicated clear glass applicator (Geuder AG, Heidelberg, Germany). The rolled graft was then implanted into the recipient’s anterior chamber through the main port. The graft was subsequently stretched in the anterior chamber using an AC tap technique by manipulating tools on the outside of the cornea which cause fluid fluctuations in the anterior chamber. After ensuring proper orientation and centralization, the graft was pressed against the recipient’s stroma by injecting sulphur hexafluoride (SF6) gas into the anterior chamber. The port wound was closed with a 10–0 nondissolvable suture. In all patients, the corneoscleral suture and dressing lens were removed 7 days after the procedure. Patients were advised to maintain a supine position until their initial follow-up visit. One-week post-surgery, patients were administered with antibiotic drops (levofloxacin) four times daily and a preservative-free topical steroid (dexamethasone sodium phosphate) eight times daily for the first month, with the dosage gradually reduced during follow-up visits.

Follow-up examinations were conducted at 7 days and then at 1, 3, 6, 12, 24, and 36 months after the procedure. During the follow-up, each patient underwent a physical examination using a slit lamp (SL-2G, Tomey, Nagoya, Japan) and a noncontact pressure monitor (iCare, IC-100, Topcon). Each time, all acquisition parameters were measured using the same equipment under consistent lighting conditions without applying pressure to the eyeball. Best corrected visual acuity was assessed using Snellen charts (Remote-Controlled Chart Monitor, CC-100, Topcon, Tokyo, Japan). Parameters of the anterior segment, such as central corneal thickness (CCT, [µm]), mean keratometry (MK, [D]), mean astigmatism (MA, [D]), astigmatism asymmetry (AA, [D]), and higher-order aberrations (HOA, [D]), were measured and calculated using anterior segment optical coherence tomography (AS-OCT), acquired with the CASIA2 instrument (Tomey, Nagoya, Japan) at 12, 24, and 36 months after surgery. Accordingly, endothelial cell density analysis was performed using an EM-4000 specular microscope (Tomey, Nagoya, Japan). In situations of uncertainty, we employed confocal microscopy to verify the corneal endothelial cell count (HRT3-RCM, Heidelberg Engineering, Heidelberg, Germany).

Statistical analysis of the results was performed using Statistica 13.1 (StatSoft, Tulsa, OK, USA), with a significance level of *p* ≤ 0.05. The normality of distributions was determined using the Shapiro–Wilk test. Since most quantitative variables had distributions that were significantly different from normal, nonparametric tests were applied. The Wilcoxon signed-rank test was used to compare the preoperative and postoperative values. Correlations between baseline variables and corneal parameters were analysed using Spearman’s rank correlation coefficient (Rs). Associations with *p* values < 0.05 were considered statistically significant.

## 3. Results

### 3.1. Characteristics of the Study Group

Thirty-four eyes from twenty-nine patients diagnosed with Fuchs’ endothelial dystrophy (FED) were included in the study and underwent DMEK surgery. The mean patient age was 68.18 ± 10.19 years. The study cohort comprised 11 men and 23 women. Among the included eyes, 33 were pseudophakic, while one remained phakic. The median graft diameter was 6.5 mm (IQR = 0.25 mm). There was also one allograft rejection case in the sixth month, which was successfully mitigated with intensified topical steroids. In the early postoperative phase elevation of the intraocular pressure occurred in three eyes (8.82%), requiring gas release in two patients (5.88%). The main complication in the late postoperative period was lens calcification, which was noted in five patients (14.7%) and could have been related to the type of material used in the intraocular lens (IOL). In nine eyes (26.47%), vision-limiting preoperative (e.g., amblyopia (n = 2), cataract (n = 1), and intraocular lens glistening (n = 1)) and postoperative comorbidities (e.g., cystoid macular oedema (n = 1), diabetic retinopathy (n = 1), epiretinal membrane (n = 1), and glaucomatous optic neuropathy (n = 1)) were reported. We did not exclude these patients from the study, as our intention was to evaluate the effectiveness of DMEK under clinical conditions that reflect real-world patient populations.

### 3.2. Changes in BCVA Recordings

**Figure 1:**
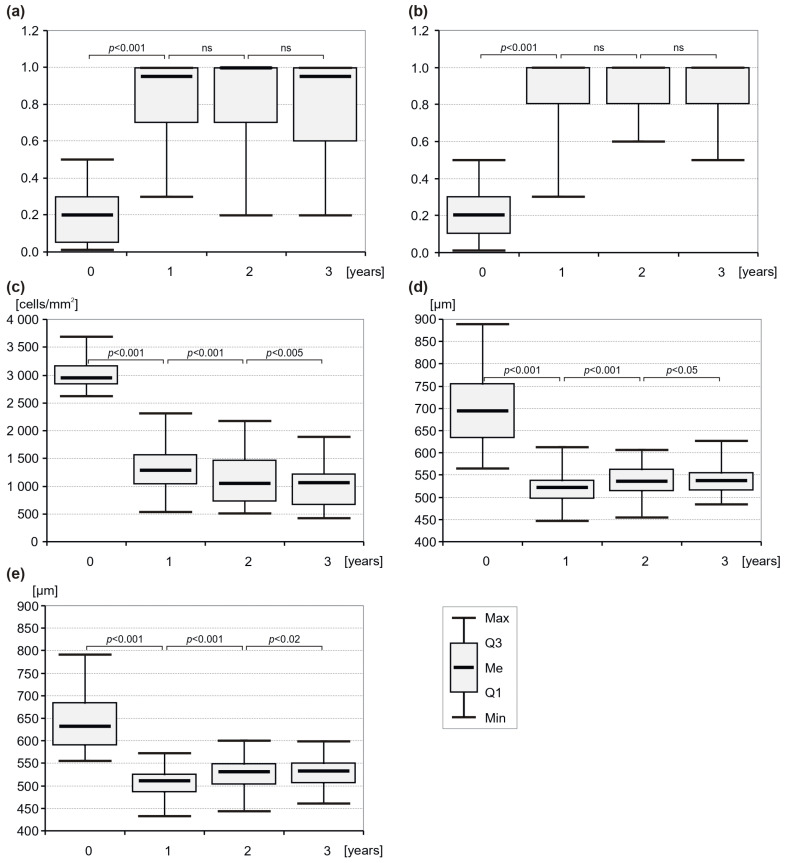
Boxplots showing (**a**) best-corrected visual acuity (BCVA) of patients with comorbidities, (**b**) BCVA of patients without comorbidities, (**c**) endothelial cell density (ECD), (**d**) central corneal thickness (CCT), and (**e**) corneal thinnest thickness (CTT) at baseline and at the first, second and third year after DMEK surgery. ns = not significant.

The impact of DMEK on BCVA over the 3-year follow-up period is presented in Figure 1a,b. In patients with ocular comorbidities (n = 9), the median (IQR) BCVA significantly improved from 0.2 (0.25) at baseline to 0.95 (0.3) at 12 months. This improvement was maintained at 24 months (1.0, IQR = 0.3, *p* = 0.67 compared with 12 months) and at 36 months (0.95, IQR = 0.4, *p* = 0.61 compared with 24 months). When eyes with ocular comorbidities limiting visual outcome after DMEK were excluded from the analyses, the BCVA reached a median of 1.0 (IQR = 0.2), with *p* < 0.001 for each time point (Figure 1b).

### 3.3. Changes in the ECD and Corneal Thickness Profile

The median (IQR) ECD significantly decreased from 2945.5 (332) cells/mm^2^ preoperatively to 1282 (531) cells/mm^2^ at 12 months (*p* < 0.0001), marking a median ECL of 56.5% in the first postoperative year. This initial decrease corresponded to a loss of 1730 (878) cells/mm^2^.

By 24 months, the ECD further declined to 1043.5 (745) cells/mm^2^, representing a median annual loss of 129 (193.84) cells/mm^2^ during the second year. At 36 months, ECD stabilised at 1062 (558) cells/mm^2^, with a median annual decline of 75 (265) cells/mm^2^ in the third year, corresponding to a cumulative ECL of approximately 63.95% compared with donor values (Figure 1c).

These changes in ECD at both the 2-year and 3-year postoperative time points relative to the first year were statistically significant (all *p* < 0.0001), indicating a consistent, though diminishing, trend in ECD beyond the first postoperative year.

Importantly, a negative correlation was observed between donor ECD and postoperative changes in recipient ECD, demonstrating that higher donor cell density was associated with a lower rate of endothelial cell loss. These correlations were statistically significant at 12 months (Rs = −0.69, *p* <0.001), 24 months (Rs = −0.67, *p* <0.001), and 36 months (Rs = −0.71, *p* < 0.001).

The CCT values decreased from 693 (122) μm (median (IQR)) preoperatively to 521.5 (42) μm after 12 months (*p* < 0.001) and then slightly but significantly increased to 534.5 (49) μm after 24 months (*p* < 0.001 compared with 12-month outcomes) and to 536.5 (41) μm after 36 months (*p* = 0.02 compared with 24-month outcomes) (Figure 1d). A similar pattern of changes was observed with regard to CTT. The median (IQR) baseline CCT was 632.0 (92) μm, and it decreased significantly by the 12-month follow-up visit (median (IQR): 511 (41) μm; *p* < 0.001). Then, it slightly but significantly increased to the median (IQR) 530 (46) μm at 24 months (*p* < 0.001, compared with the 12-month values), with a median (IQR) 531.5 (44.5) μm at 36 months (*p* = 0.01 compared with the 24-month values) (Figure 1e).

Importantly, we found no correlation between corneal thickness characteristics and the dynamics of the decreased endothelial cell count at any follow-up visit.

### 3.4. Changes in Corneal Topography Parameters

Furthermore, we analysed the dynamics of changes in topographic parameters, such as keratometry, corneal astigmatism, astigmatism asymmetry, and high-order aberrations, at baseline and at consecutive follow-up time points after DMEK surgery. Table 1 shows the variations in the corneal parameters for the 3 and 6 mm optical zones (OZ).

The median baseline MK of the anterior surface was 49.65 D for the 3 mm OZ and 49.28 D for the 6 mm OZ and remained unchanged at the 36th month postoperatively (median = 48.79; *p* = 0.2 and median = 48.60; *p* = 0.31, respectively). Similarly, we observed no significant changes in total keratometry in either zone between baseline recordings (median = 43.62 D for 3 mm OZ and median = 43.41 D for 6 mm OZ) and 36-month data (median = 43.98 D for 3 mm OZ and median = 43.66 D for 6 mm OZ, *p* = 0.32 and *p* = 0.31, respectively). With respect to the posterior corneal surface, we observed a significant reduction in keratometry values at the 36th month compared with baseline values, indicating steepening of the posterior corneal curvature.

The median preoperative anterior astigmatism was 1.39 for the 3 mm OZ. At 12 months post-surgery, these values changed to 0.87 (*p* = 0.02) and remained stable at the 24-month follow-up visit (*p* = 0.10 compared with the 12-month values) and at the 36-month follow-up visit (*p* = 0.93 compared with the 24-month outcomes). In contrast, anterior astigmatism in the 6 mm OZ did not significantly change at 12 months compared with baseline values (*p* = 0.08) and did not change at 24 months (*p* = 0.07 compared with the 12-month values) and 36 months (*p* = 0.56 compared with the 24-month values).

We observed a significant reduction in posterior astigmatism values during the first 12 months of observation compared with baseline values for both optical zones (*p* < 0.001 for 3 mm OZ and *p* < 0.001 for 6 mm Oz). The parameters for the 3 mm Oz remained stable at the 24-month and 36-month follow-up visits. The parameters for 6 mm Oz decreased significantly in the second observation period (*p* = 0.01 compared with the 12-month value), and henceforth stabilised.

Overall, at 36 months, the total astigmatism magnitude was significantly lower than the baseline outcomes for both the 3 mm and the 6 mm OZ (*p* = 0.001 for the 3 mm OZ and *p* = 0.003 for the 6 mm OZ). When the sequential changes at successive time points were analysed, the total astigmatism power significantly decreased between baseline and the 12th month (*p* = 0.01 for 3 mm OZ and *p* = 0.07 for 6 mm OZ) and subsequently decreased between the 12th and 24th months (*p* = 0.04 for 3 mm OZ and *p* = 0.02 for 6 mm OZ), with consecutive stabilisation of the astigmatism power from the 24th month up to the 36th month of follow-up (*p* = 0.89 for 3 mm OZ and *p* = 0.84 for 6 mm OZ). Interestingly, the axis of the baseline total astigmatism remained unchanged throughout the entire follow-up period.

Next, we evaluated irregular corneal astigmatism with asymmetry of the astigmatic components (Table 1). We observed a significant reduction in astigmatic asymmetry for all corneal surfaces in the first 12 months of observation (median and *p* compared with the baseline: anterior: 1.0 D vs. 0.44 D (*p* < 0.001) for 3 mm OZ and 1.29 D vs. 0.64 D (*p* < 0.001) for 6 mm OZ, posterior: 0.29D vs. 0.13 D (*p* < 0.001) for 3 mm OZ and 0.32 D vs. 0.13 D (*p* < 0.001) for 6 mm OZ, total: 1.32 D vs. 0.46 D (*p* < 0.001) for 3 mm OZ and 1.74 D vs. 0.63 D (*p* < 0.001) for 6 mm OZ). Thereafter, the astigmatism asymmetry parameters stabilised until the 36th month.

Next, we analysed the higher-order corneal aberration variations (Table 1). A significant HOA reduction in the anterior and posterior HOA after the procedure was observed at 12 months post-surgery (anterior cornea: *p* < 0.001 for 3 mm OZ and *p* < 0.001 for 6 mm OZ; posterior cornea: *p* < 0.001 for 3 mm OZ and *p* < 0.001 for 6 mm OZ), whereas the values remained stable at 24 months (anterior cornea: *p* = 0.98 for 3 mm OZ and *p* = 0.54 for 6 mm OZ; posterior cornea: *p* = 0.74 for 3 mm OZ and *p* = 0.62 for 6 mm OZ) and remained unchanged at 36 months compared with values at 24 months post-surgery (anterior cornea: *p* = 0.64 for 3 mm OZ and *p* = 0.48 for 6 mm OZ; posterior cornea: *p* = 1.0 for 3 mm OZ and *p* = 0.38 for 6 mm OZ). A similar pattern of changes was observed for the total corneal readings. At 12 months postsurgery, there was a statistically significant decrease compared with baseline values (*p* < 0.001) for the 3 mm OZ group and *p* < 0.001 for the 6 mm OZ group. We did not observe changes in total HOA between the 12th and 24th months (*p* = 0.46 for 3 mm OZ and *p* = 0.79 for 6 mm OZ) and between the 24th month and 36th month of follow-up (*p* = 0.61 for 3 mm OZ and *p* = 0.41 for 6 mm OZ).

### 3.5. Impact of Corneal Graft Diameter on Corneal Parameter Changes

Furthermore, we analysed the correlations between graft diameter and changes in corneal parameters at the follow-up time points postoperatively. We noted no influence of graft diameter on VA, ECD, and CCT changes at any time point of observation. Interestingly, we found that larger grafts resulted in smaller changes in total keratometry in both zones between baseline recordings and 12-month data (Rs = −0.28, *p* = 0.11 for 3 mm OZ and Rs = −0.35, *p* = 0.04 for 6 mm OZ), 24-month data (Rs = −0.37, *p* = 0.04 for 3 mm OZ and Rs = −0.38, *p* = 0.04 for 6 mm OZ) and 36-month data (Rs = −0.28, *p* = 0.12 for 3 mm OZ and Rs = −0.26, *p* = 0.14 for 6 mm OZ). We also observed positive correlations between graft diameter and posterior astigmatism magnitude changes. This means that the larger the graft is, the greater the change in posterior astigmatism compared with the baseline outcomes at 12 months (Rs = 0.36, *p* = 0.03 for 3 mm OZ and Rs = 0.39, *p* = 0.02 for 6 mm OZ), 24 months (Rs = 0.35, *p* = 0.055 for 3 mm OZ and Rs = 0.44, *p* = 0.013 for 6 mm OZ) and 36 months (Rs = 0.45, *p* = 0.009 for 3 mm OZ and Rs = 0.53, *p* = 0.002 for 6 mm OZ) postoperatively.

### 3.6. Changes in Corneal Parameters in Relation to Baseline Values

Further analysis revealed that the initial preoperative values of corneal parameters strongly influenced the patterns of their changes after DMEK surgery. Specifically, we identified significant negative correlations between baseline levels of CCT, keratometry, astigmatism, astigmatism asymmetry, and higher-order aberrations (HOAs) and their changes during postoperative follow-up, which lasted up to 36 months. These findings suggest that a thicker cornea and greater preoperative values of keratometry, astigmatism, astigmatism asymmetry, and HOA are associated with less pronounced reductions in these parameters after surgery (Table 2).

Additionally, our findings revealed a negative correlation between preoperative central corneal thickness (CCT) and postoperative changes in various corneal topographic parameters, including CCT, astigmatism, astigmatism asymmetry, and higher-order aberrations (HOAs) (Table 3). These findings suggest that the thicker the preoperative cornea is, the smaller the reduction in both regular and irregular corneal astigmatism and HOAs following surgery.

We also found better BCVA preoperatively, with a greater CCT decrease compared to baseline values at consecutive follow-up visits after DMEK surgery, e.g., at 12 months (Rs = 0.49, *p* =0.003), 24 months (Rs = 0.41, *p* = 0.024), and 36 months (Rs = 0.41, *p* = 0.019).

## 4. Discussion

There is no clear evidence of an optimal graft size for DMEK in the available literature. Various graft sizes were used by different study groups, some of which had a constant shape, and others were more flexible based on the recipient requirements. Indeed, most grafts were punched between 8 and 8.75 mm [5,6,8,9,10,11], whereas many authors did not specify the graft size used [3,12,13,14,15,16,17].

Hemi-DMEK using two half-moon-shaped grafts has been introduced to double the yield of transplants from the same donor pool [18]. The utility of small-diameter 4 mm circular grafts for treating central endothelial disease has been documented in preclinical studies [19]. Accordingly, quarter-DMEK grafts have been proposed as a feasible procedure to increase the availability of endothelial grafts with visual outcomes similar to those of conventional circular DMEK grafts [4].

In the present study, we used a circular graft with a median diameter of 6.5 mm. To the best of our knowledge, no studies have used this graft diameter in the treatment of FECD.

Our findings indicate that near-normal visual acuity can be achieved and sustained over a three-year follow-up period, which aligns with the results of previous studies reporting stable visual acuity in long-term assessments of DMEK procedures [9,14]. Publications addressing extended follow-up periods indicate that high visual acuity can persist for up to a decade [20,21,22].

Notably, in our analysis, significant improvement in best-corrected visual acuity (BCVA) was observed across both groups, those without comorbidities and those with coexisting conditions. Nonetheless, patients without comorbidities demonstrated more stable long-term improvement, this finding is consistent with results of other studies that have categorised patients similarly [11,21].

With respect to ECD, we observed constant endothelial cell loss. The initial postoperative ECL observed in this study was markedly high. Over time, the median (IQR) ECL rate decreased by 129 (193.84) cells/mm^2^ in the second year and 75 (265) cells/mm^2^ in the third year.

Our findings align with previous mid- and long-term studies on Descemet membrane endothelial keratoplasty (DMEK), demonstrating an initial endothelial cell loss (ECL) that subsequently stabilises, albeit with variations in specific decline rates. For instance, Birbal et al. documented a 40% reduction in ECL within the first year, followed by a sustained annual decrease of approximately 96.4 cells/mm^2^ (7%) [14]. Similarly, Ham et al. observed a consistent annual decline of 115 cells/mm^2^ (9.5%) over a seven-year period [20]. Baydoun et al. reported a persistent ECL of 92.6% cells/mm^2^ (7%) per year across five years, whereas Vasiliauskaitė et al. documented an annual loss of 115 cells/mm^2^ (8%) over ten years, with a progressive deceleration in the rate of decline [22,23]. Furthermore, Schlögl et al. described an initial 42.93% reduction in the first postoperative year, followed by continued endothelial cell loss during the second and third years of follow-up [11]. Notably, their findings also indicated a slight increase in endothelial cell density (ECD) during the fourth and fifth years.

These observations underscore a generalised trend of ECL stabilisation over time, though the rate of decline remains influenced by multiple factors, including surgical technique, endothelial cell density (ECD) measurement methodologies, and postoperative management protocols.

Thus, while the initial ECL may vary, our findings reinforce the general trend of stabilisation after the first year, supporting the durability of DMEK.

The CCT has been widely used as an index of graft function. Our data provided evidence that CCT readings decreased in the first 12 months after the operation compared with those recorded preoperatively. However, the continuous increase in CCT in our study after the second and third years may indicate endothelial cell loss and redistribution/migration over the graft and stroma. These observations are consistent with those in the majority of reports [11,22,23,24].

In their long time-course analysis, Bichet et al. showed that the CCT decreased immediately after surgery and then increased steadily over time, with an annual average of 16 μm [9]. Importantly, we observed that the baseline CCT plays a key role in influencing variations in corneal thickness after surgery. We identified a strong negative correlation between the initial CCT and changes in corneal thickness during follow-up postoperative visits. Thus, the preoperative CCT represents an efficient predictor of relevant outcome parameters after DMEK surgery [24]. Indeed, Brockmann et al. indicated that DMEK should be performed in eyes with Fuchs’ endothelial corneal dystrophy, if possible, before the CCT exceeds 625 µm to maintain good clinical results [25].

We also found that better BCVA preoperatively was associated with a greater decrease in CCT postoperatively. This is supported by previous studies showing that patients with poor baseline BCVA recover less well and more slowly than patients with better BCVA [26].

We also provided detailed characteristics of the changes in corneal topography within 36 months of observation. Importantly, most studies have focused on short-term changes in refractive and topographic parameters up to 1 year after DMEK, with only a few addressing longer follow-up periods [27,28,29]. A literature review by Deng and colleagues emphasised that among the analysed studies, only two had a follow-up period exceeding 24 months, highlighting the limited availability of long-term data in this field [30]. The extended observation period in our study allows for a more accurate evaluation of the long-term refractive and topographic stability following DMEK.

Our findings demonstrated a reduction in posterior keratometry measurements compared with preoperative values, indicating increased steepness of the posterior corneal curvature. In contrast, anterior corneal keratometry remained largely unchanged. Importantly, these changes did not significantly affect the total keratometry score at the 36-month follow-up. The overall changes from baseline to 36 months were minimal (median: −0.36 D in the 3 mm OZ and −0.25 D in the 6 mm OZ) and statistically insignificant (*p* > 0.05).

The magnitude of keratometric changes secondary to DMEK surgery and the timeline varied across studies. Chamberlain et al. reported stabilisation as early as six months post-DMEK, with no further changes by 24 months in the 3 mm OZ [27]. In contrast, van Dijk et al. reported greater posterior steepening than that in our study, with significant variability [29]. Agha et al. also reported a greater steepening magnitude but concluded that hyperopic changes are minor and manageable, with no significant effect on IOL calculations [12]. Notably, they highlighted that pseudophakic eyes experience less pronounced hyperopia than combined cataract and DMEK surgeries. Hayashi et al. reported slightly greater posterior steepening than our results but noted no influence on total MK or hyperopic shift, emphasising the minimal impact on IOL power calculations [10].

Our study provides a long-term perspective on changes in astigmatism following DMEK. The results indicate that surgical changes have a minimal impact on the orientation of the astigmatism axis, whereas the total astigmatism power significantly decreased over the longer observation period. Shajari M et al. recommend avoiding simultaneous implantation of toric IOLs in “triple” procedures or postponing toric IOL implantation until corneal parameters stabilise to minimise refractive errors [16]. Our study, which incorporated a longer observation period, revealed that corneal astigmatism stabilised within a 24-month follow-up period, thus providing additional insights into long-term refractive stability.

Our study demonstrated that a higher-order aberration (HOA) decrease primarily occurred within the first 12 months after surgery, followed by stability, which persisted throughout the 36-month observation period.

Hayashi et al. reported reductions in posterior and anterior HOAs by 12 months, although values remained higher than those in controls [10]. Lin et al. reported significant reductions in posterior HOAs by 24 months, which is consistent with our findings of stabilisation by 12 months [31]. Overall, most studies confirm the efficacy of DMEK in reducing HOAs and enhancing optical quality. However, our extended 36-month follow-up uniquely highlights the long-term durability and sustained stability of these improvements.

A key limitation of this study is the lack of a control group for the direct comparison of outcomes with alternative graft sizes. However, our decision to focus solely on the 6.5 mm graft diameter was guided by the extensive body of literature that primarily investigates larger graft diameters (typically between 8.0 and 8.75 mm). These studies provide well-documented reference points regarding endothelial survival, visual outcomes, and refractive stability, allowing for indirect comparisons of our findings with previously established data.

Additionally, while our study presents a comprehensive three-year follow-up, longer observation periods would be beneficial to further assess the long-term endothelial cell survival and corneal stability associated with smaller-diameter DMEK grafts. Another limitation of this study is the relatively small sample size (34 patients); however, it should be noted that the study is ongoing, and this number represents only those patients who have currently completed the full 36-month follow-up. As more patients reach this milestone, further data analysis will enable a more comprehensive evaluation of the outcomes.

Moreover, variability in the initial donor endothelial cell density (ECD) may influence long-term endothelial survival and graft function. Higher ECD values in donor tissues have been associated with a lower rate of postoperative endothelial cell loss, potentially affecting the longevity of the graft. Although our study does not specifically stratify patients based on donor ECD, future research should investigate this parameter as a potential predictor of graft survival.

Another important consideration is the long-term graft adherence in small-diameter DMEK. While smaller grafts may have a lower rebubbling rate due to improved adherence, long-term data on graft stability and detachment risk are still limited. The role of graft size in determining rebubbling rates, endothelial survival, and overall surgical success warrants further investigation through longitudinal, controlled studies.

Future studies comparing different graft sizes within a prospective controlled trial would provide more definitive conclusions regarding the optimal balance between graft size, endothelial cell survival, and graft adherence.

## 5. Conclusions

The use of smaller grafts in DMEK demonstrates comparable postoperative outcomes to those achieved with larger grafts, highlighting their feasibility and effectiveness in maintaining high visual and refractive quality while offering potential advantages in tissue.

## Figures and Tables

**Table 1:** The changes in mean keratometry, astigmatism magnitude, astigmatism asymmetry, and higher-order aberration power between the follow-up time points in two different corneal optical zones after Descemet’s membrane endothelial keratoplasty.

			Baseline Median (IQR)	12th Month Median (IQR)	24th Month Median (IQR)	36th Month Median (IQR)	*p* *
Mean keratometry [D]	Anterior	3 mm OZ	49.65 (4.14)	48.81 (2.39)	48.44 (2.58)	48.79 (2.41)	0.19
		6 mm OZ	49.28 (2.86)	48.37 (2.22)	48.13 (2.36)	48.60 (2.41)	0.31
	Posterior	3 mm OZ	6.01 (0.68)	6.32 (0.31)	6.30 (0.37)	6.33 (0.51)	**0.001**
		6 mm OZ	5.97 (0.79)	6.30 (0.30)	6.28 (0.32)	6.30 (0.51)	**0.001**
	Total	3 mm OZ	43.62(3.77)	43.16 (2.67)	43.48 (2.31)	43.98 (2.38)	0.31
		6 mm OZ	43.41 (3.05)	42.87 (2.20)	43.21 (2.12)	43.66 (2.06)	0.10
Mean astigmatism [D]	Anterior	3 mm OZ	1.39 (1.20)	0.87 (0.73)	0.87 (0.76)	0.86 (0.63)	**0.01**
		6 mm OZ	1.13 (1.00)	0.82 (0.52)	0.75 (0.61)	0.90 (0.64)	**0.06**
	Posterior	3 mm OZ	0.26 (0.29)	0.16 (0.13)	0.16 (0.10)	0.16 (0.13)	**<0.00**
		6 mm OZ	0.23 (0.26)	0.14 (0.10)	0.13 (0.09)	0.14 (0.11)	**<0.00**
	Total	3 mm OZ	1.30 (1.61)	0.83 (0.55)	0.78 (0.68)	0.77 (0.61)	**0.001**
		6 mm OZ	1.10 (1.00)	0.74 (0.48)	0.67 (0.55)	0.77 (0.63)	**0.003**
Astigmatism asymmetry [D]	Anterior	3 mm OZ	1.00 (1.81)	0.44 (0.31)	0.40 (0.48)	0.34 (0.27)	**<0.00**
		6 mm OZ	1.29 (2.33)	0.64 (0.42)	0.58 (0.50)	0.49 (0.41)	**<0.00**
	Posterior	3 mm OZ	0.29 (0.52)	0.13 (0.13)	0.13 (0.13)	0.13 (0.10)	**<0.00**
		6 mm OZ	0.32 (0.45)	0.13 (0.11)	0.14 (0.14)	0.16 (0.09)	**<0.00**
	Total	3 mm OZ	1.32 (1.27)	0.46 (0.39)	0.36 (0.44)	0.31 (0.26)	**<0.00**
		6 mm OZ	1.74 (1.62)	0.63 (0.42)	0.52 (0.45)	0.48 (0.35)	**<0.00**
Higher-order aberrations [D]	Anterior	3 mm OZ	0.43 (1.04)	0.21 (0.09)	0.21 (0.13)	0.21 (0.08)	**<0.00**
		6 mm OZ	0.46 (0.82)	0.29 (0.14)	0.30 (0.13)	0.28 (0.16)	**0.001**
	Posterior	3 mm OZ	0.15 (0.16)	0.07 (0.04)	0.06 (0.03)	0.06 (0.05)	**<0.00**
		6 mm OZ	0.16 (0.12)	0.08 (0.04)	0.08 (0.04)	0.07 (0.05)	**<0.00**
	Total	3 mm OZ	0.43 (0.96)	0.20 (0.09)	0.19 (0.11)	0.19 (0.08)	**<0.00**
		6 mm OZ	0.50 (0.86)	0.26 (0.13)	0.27 (0.12)	0.25 (0.15)	**<0.00**

Statistically significant values are shown in bold. * *p* values are calculated for intervals between baseline and 36th month.

**Table 2:** The correlations between changes in VA, CCT, keratometry, astigmatism, astigmatism asymmetry, and higher-order aberration and baseline values of those parameters.

Correlation			Change in the Selected Parameter at 12th Month as Compared to Baseline	Change in the Selected Parameter at 24th Month as Compared to Baseline	Change in the Selected Parameter at 36th Month as Compared to Baseline
Baseline BCVA			−0.30	−0.16	**−0.40**
Baseline CCT			**−0.89**	**−0.94**	**−0.90**
Mean keratometry [D]	Anterior	3 mm OZ	**−0.76**	**−0.77**	**−0.79**
		6 mm OZ	**−0.70**	**−0.68**	**−0.68**
	Posterior	3 mm OZ	**−0.82**	**−0.82**	**−0.75**
		6 mm OZ	**−0.79**	**−0.78**	**−0.74**
	Total	3 mm OZ	**−0.84**	**−0.80**	**−0.79**
		6 mm OZ	**−0.81**	**−0.80**	**−0.74**
Mean astigmatism [D]	Anterior	3 mm OZ	**−0.79**	**−0.88**	**−0.79**
		6 mm OZ	**−0.67**	**−0.74**	**−0.59**
	Posterior	3 mm OZ	**−0.82**	**−0.93**	**−0.86**
		6 mm OZ	**−0.80**	**−0.90**	**−0.82**
	Total	3 mm OZ	**−0.81**	**−0.90**	**−0.85**
		6 mm OZ	**−0.78**	**−0.80**	**−0.74**
Astigmatism asymmetry [D]	Anterior	3 mm OZ	−0.10	−0.06	−0.16
		6 mm OZ	−0.20	−0.01	−0.10
	Posterior	3 mm OZ	**−0.93**	**−0.92**	**−0.87**
		6 mm OZ	**−0.90**	**−0.87**	**−0.82**
	Total	3 mm OZ	**−0.89**	**−0.89**	**−0.98**
		6 mm OZ	**−0.92**	**−0.87**	**−0.97**
Higher-order aberrations [D]	Anterior	3 mm OZ	**−0.91**	**−0.95**	**−0.89**
		6 mm OZ	**−0.92**	**−0.94**	**−0.87**
	Posterior	3 mm OZ	**−0.93**	**−0.93**	**−0.93**
		6 mm OZ	**−0.91**	**−0.93**	**−0.91**
	Total	3 mm OZ	**−0.95**	**−0.96**	**−0.92**
		6 mm OZ	**−0.93**	**−0.97**	**−0.91**

Statistically significant values are shown in bold. *p* values were calculated for intervals relative to preoperative values.

**Table 3:** The correlations between baseline CCT and the changes in VA, CCT, keratometry, astigmatism, astigmatism asymmetry, and higher-order aberration values obtained in the 3- and 6 mm optical zones after DMEK surgery.

Correlation of the Baseline CCT and:			At Baseline	At 12th Month	At 24th Month	At 36th Month
BCVA			**−0.40**	0.12	0.02	0.23
CCT			1.00	**−0.88**	**−0.94**	**−0.90**
ECD			−0.002	−0.17	−0.05	−0.04
Mean keratometry [D]	Anterior	3 mm OZ	−0.07	−0.13	−0.10	−0.04
		6 mm OZ	−0.19	−0.06	−0.06	−0.01
	Posterior	3 mm OZ	−0.20	0.20	0.19	0.18
		6 mm OZ	−0.24	0.23	0.21	0.24
	Total	3 mm OZ	−0.04	−0.13	−0.13	−0.13
		6 mm OZ	−0.08	−0.02	−0.12	−0.11
Mean astigmatism [D]	Anterior	3 mm OZ	**0.35**	**−0.37**	−0.34	−0.32
		6 mm OZ	**0.34**	**−0.38**	−0.34	−0.31
	Posterior	3 mm OZ	**0.57**	**−0.60**	**−0.62**	**−0.51**
		6 mm OZ	**0.61**	**−0.61**	**−0.62**	**−0.58**
	Total	3 mm OZ	**0.47**	**−0.44**	**−0.42**	−0.32
		6 mm OZ	**0.47**	**−0.51**	**−0.44**	−0.31
Astigmatism asymmetry [D]	Anterior	3 mm OZ	**0.55**	−0.08	−0.11	0.03
		6 mm OZ	**0.48**	−0.09	−0.03	−0.06
	Posterior	3 mm OZ	**0.42**	**−0.39**	−0.33	**−0.39**
		6 mm OZ	**0.42**	**−0.43**	−0.35	**−0.40**
	Total	3 mm OZ	**0.57**	**−0.55**	**−0.57**	**−0.54**
		6 mm OZ	**0.49**	**−0.51**	**−0.55**	**−0.46**
Higher-order aberrations [D]	Anterior	3 mm OZ	**0.39**	**−0.49**	**−0.49**	−0.30
		6 mm OZ	**0.36**	**−0.45**	**−0.45**	**−0.32**
	Posterior	3 mm OZ	**0.69**	**−0.64**	**−0.70**	**−0.65**
		6 mm OZ	**0.71**	**−0.65**	**−0.69**	**−0.60**
	Total	3 mm OZ	**0.49**	**−0.55**	**−0.55**	**−0.40**
		6 mm OZ	**0.44**	**−0.50**	**−0.49**	**−0.40**

Correlations were calculated for three consecutive time points, i.e., 12, 24, and 36 months postoperatively. At the zero-observation time point, baseline CCT values refer to baseline absolute values of listed parameters. Significant values are shown in bold.

## Data Availability

The data used to support the findings of this study are available from the corresponding author upon request.
